# Environmental Pollution and Related Hazards at Agbara Industrial Area, Ogun State

**DOI:** 10.1038/s41598-018-24810-4

**Published:** 2018-04-24

**Authors:** Ojekunle Z. O., Jinadu O. O. E., Afolabi T. A., Taiwo A. M.

**Affiliations:** 1 Department of Environmental Management and Toxicology, College of Environmental Resources Management, Federal University of Agriculture Abeokuta, Abeokuta, Ogun state Nigeria; 2 Department of Chemistry, College of Physical Sciences, Federal University of Agriculture Abeokuta, Abeokuta, Ogun state Nigeria

## Abstract

This study aimed at assessing the environmental pollution and related hazards of industries at Agbara, Ogun State, Nigeria. A total of five sampling points were identified and selected at random. Environmental samples were collected on a weekly basis for duration of 10 weeks. Air pollutants measured were CO_2_, CO, NO, NO_x_, VOCs, H_2_S, SO_2_, NH_3_, PM_2.5_ andPM_10_ using standard procedure. Dust and plant samples were also collected and analyzed for heavy metals (Pb, Cr, Cd, Cu and Zn) using the Atomic Absorption Spectrophotometer (AAS). Data was evaluated for descriptive and inferential statistics using SPSS for Windows version 22.0. Air pollution data were also subjected to SPE-risk model. The results of highest measured air parameters were: CO (5.50 ± 2.32 ppm), CO_2_ (3.00 ± 2.05%), NO_x_ (0.90 ± 0.32 ppm), NO (0.60 ± 0.52 ppm), PM_10_ (0.40 ± 0.52 mg/m^3^) and PM_2.5_ (0.20 ± 0.42 mg/m^3^). The results of heavy metal concentrations in dust samples were: 57.40 ± 13.28 mg/kg for Cu, 45.36 ± 12.37 mg/kg for Cr, 22.80 ± 17.36 mg/kg for Zn, 13.76 ± 3.08 mg/kg for Pb and 0.32 ± 0.36 mg/kg for Cd. Metal concentrations in plants were: Cu (70.07 ± 16.24 mg/kg), Zn (67.69 ± 14.50 mg/kg), Cr (22.46 ± 9.35 mg/kg), Pb (13.76 ± 3.08 mg/kg) and Cd (2.25 ± 3.04 mg/kg). This study revealed the concentrations of CO_2_, NO_x_ and NO higher than the World Health Organization (WHO) permissible standards while Pb, Cu, Cr, Cd and Zn values in dust samples were also found above the National Environmental Standards and Regulations Enforcement Agency (NESREA) and the WHO standards. Results of SPE-RISK model indicated that CO_2_, CO, Pb, Cu and Zn posed the greatest health risks, while the Principal Component Analysis (PCA) indentified pollutant sources from industrial and vehicle exhaust.

## Introduction

Lithosphere and hydrosphere are natural resources that enhance agriculture and development of human race but are frequently subjected to great exploitation and degradation by natural and human factors^1^. McGrath *et al*.^2^ opined two kind of pollution sources are responsible for these degradation of land and water which include pollution from industrialization, agricultural activities and motorization usage while each of the sources affect plants and animals alike as well as posing great danger to human health. Most heavy metals are not easily degradable and thus find their ways into water body and soils, where they are bio-accumulated in food chain. Human are exposed to certain carcinogenic metals (As, Cd, Ni, Pb, Cr and Co) through ingestion of contaminated foods^3^. These metals thereby, pose great threat to human existence and the environment at large.

Exposure to environmental pollutants in the cities is of severe environmental and health concerns today in the world^4^. Studies have documented linkages between health effects and atmospheric pollution^5^ leading to serious health effects ranging from respiratory related diseases to chronic diseases that could lead to high mortality^6^. Bell *et al*.^7^ opined that poor hygiene and health care delivery are related to low income populations who are exposed to atmospheric pollution, because they live in blighted environment, industrial areas and roadsides to gain access to work place. They thereby exposed themselves to all sorts of environmental hazards; and in many cases denied access to health care delivery leading to death^7^. Exposure is particularly high among women and young children, who spend the most time near the domestic hearth. The World Health Organization is providing technical support to countries in their own evaluations and scale up of promoting safer stove technologies, as well as air quality guidelines to offer global guidance on reducing the health impacts of air pollution^8^.

In addition, industrialization, development and economic activities had increased globally over the years as a result of man quest to improved standard of living and these are manifested in fossil fuel production and usage for motorization. Ibeto *et al*.^9^ identified petrol chemical industry as main sources environmental pollution. Pb, Cd, Ni, Mn and Cr had been largely linked to many of these disease affecting man and its environment^10^. In general, heavy metals cause systemic toxicity, influences behavior, disrupts neurological system and mental function. Ibeto and Okoye^11^, have identified heavy metals in soil and water body in large quantities as a result of industrial pollution.

Studies have shown that soils and dusts are contaminated by percolation and infiltration of heavy metals during industrialization, manufacturing, agriculture, mining, waste disposal, industrial effluents, and disposition from the atmosphere^12,13^. In most cases, metals do not degrade by microbial of chemical means^14^, and persist once been introduced to the medium^15^ and severely prevent the biodegradation or deconcentration of any organic contaminants found within its immediate environment^16^. Consequently, they pose risks and hazards to man through the ecosystem or direct ingestion, inhalation or dermal contact. McLaughlin *et al*.^17^ viewed phytotoxicity to reduce land profitability and agricultural activities with resultant effects on food security and sustainable development. It is important to carry out a research on environmental pollution and its related hazards in order to proffer mitigating measures aimed at safeguarding the environmental and public health.

## Materials and Methods

### Study Area

Agbara industrial area is a fast growing town in Ogun State. The industrial area is a model integrated Town development on 454.1 hectares of land. It is situated approximately 31 kilometers west of Lagos on the Lagos-Badagry expressway on high ground above the Owo River and derives its name from the neighbouring Agbara village^18^. It center lies at 6°31′0″ North, 3°6′0″ East (www.gomapper.com) and it has an elevation of 37 meters above sea level. The industrial areas are made up of 41.55% (188.289 hectares) of the whole estate^18^. The location and accessibility of Agbara Estate makes the transportation of raw materials and finished goods. Figure (a) shows the study area and the sampling locations.

### Sample Collection and Analysis

#### Air Measurements

**Figure 1:**
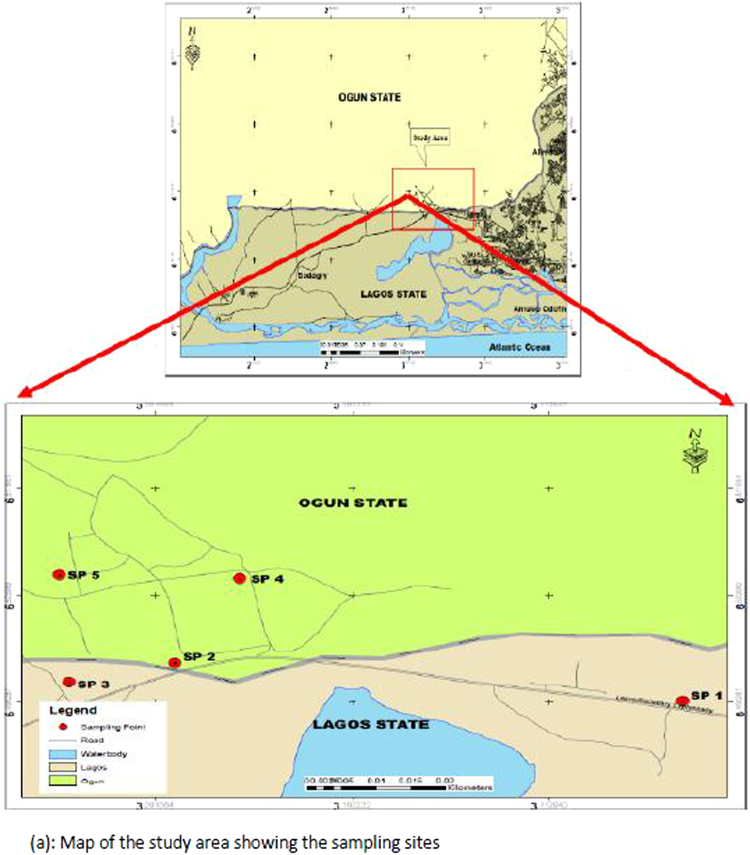
Area map of Agbara.

Air pollutant samples (gaseous and particulate matter) were collected within a 30 m radius, between the hours of 10:00 am – 2:00 pm, from 14^th^ of August – 16^th^ of October, 2015 (10 weeks) at the 5 (five) sampling points (Fig. 1). The air quality indicators were measured and monitored in unconfined (outdoor) environment at the sampling locations. The sampling equipment is presented in Table 1. All samples were taken 3 times to minimize error from device readings.

**Table 1:** Equipment and their uses.

Equipment	Readings
Air borne particulate monitor, model – PDR-1200	%CO_2_, CO, SO_2_, NO and NO_X_
Industrial Scientific iTX Multi Gas Monitor Tester iSP Sample Pump	H_2_S, NH_3_ and VOC
Thermo Scientific pDR-1000AN (Personal Data Ram)	PM_10_ and PM_2.5_
eTrex Legend H – Garmin GPS Handheld device	GPS co-ordinates

#### Dust, Plant Sampling and Preparation

Dust was collected in triplicates from the road sides of the five (5) sampling points The samples were collected with a stainless steel spoon and placed in clean polyethylene bags to avoid contamination. Each of the samples was labeled with a paper tape and a pen. The samples were sun dried for two (2) weeks.

Plant samples were collected from the sampling points in triplicates. The plant species used was spear grass (*Heteropogoncontortus* (L.) due to its relative abundance at each sampling point. Each of the samples was uprooted and placed into polyethylene bags and were labeled using paper tape and a pen.

#### Chemical Analysis for Soil and Plant

A rapid nitric‐perchloric acid digestion method was used for the dust and plant analyses. The dust and plant samples were sieved with a 0.5 mm mesh sieve. The samples were pulverized and weighed (0.5 g) into a conical flask where 10 ml mixed acids (Perchloric acid and nitric acid in ratio of 1:2) was added and allowed to undergo heating on a digester hot plate at 105 °C for about 30 minutes until the colour changed. The digested sample was allowed to cool and then filtered and made up to 50 ml with distilled water. The digested sample was read using Bulk Scientific Atomic Absorption Spectrophotometer (model 210/211 Vap) to analyze the concentrations of Pb, Cd, Cr, Cu and Zn. The blank samples were also analyzed to determine the detection limit (3 × standard deviation of six blank samples), and limit of quantification (10 × detection Limit). All samples were analyzed in triplicates for good precision and accuracy. All chemicals used for analysis were of Analytical grade.

### Statistical analysis

All data obtained from the earlier mentioned analysis were subjected to descriptive analysis, analysis of variance (ANOVA), the test of significance of the mean by Duncan’s Multiple Range (DMR) test, and Principal component Analysis (PCA) using SPSS for Windows version 16.0.

### SPE – risk analysis

The SPE is a quick and dirty Risk Assessment process that can easily be used in the field

$${\rm{Risk}}\,({\rm{R}})={\rm{Severity}}\times {\rm{Probability}}\times {\rm{Exposure}}$$

Identify specific hazards and assign them a value for each elemental pollutants mentioned above.

The higher the number, the greater the Severity, Probability or Exposure.

#### Severity

Scored 1 to 5. Describes the potential loss or consequence or mishap. Protective devices or procedures are used to mitigate Severity.

$$1={\rm{none}}\,{\rm{or}}\,{\rm{slight}}\,2={\rm{Minimal}}\,3={\rm{Significant}}\,4={\rm{Major}}\,5={\rm{Catastrophic}}$$

#### Probability

Scored 1 to 5. The likelihood that given the Exposure, the projected consequences will occur. Training situation awareness, morale and attitude change are used to mitigate Probability.

$$\begin{array}{c}1={\rm{Impossible}}\,{\rm{or}}\,{\rm{remote}}\,{\rm{under}}\,{\rm{normal}}\,{\rm{conditions}}\,2={\rm{Unlikely}}\,{\rm{under}}\,{\rm{normal}}\,{\rm{conditions}}3\\ \,\,\,=50/50\,{\rm{chance}}\,4={\rm{Greater}}\,{\rm{than}}\,50 \% \,{\rm{chance}}\,5={\rm{Very}}\,{\rm{likely}}\end{array}$$

Exposure: Scored 1 to 4. The amount of time, number of cycles, number of people and resources (equipment) involved.

$$1={\rm{None}}\,{\rm{or}}\,{\rm{below}}\,{\rm{average}}\,2={\rm{Average}}\,3={\rm{Above}}\,{\rm{average}}\,4={\rm{Great}}$$

## Results and Discussion

### Air pollutant data

The air quality data, heavy metals in dust and plants at the five (5) different locations within Agbara industrial district are shown in Tables 2–4. Table 2 shows the concentrations of various air pollutants at the sampling sites. The highest concentrations of CO (5.50 ± 2.32 ppm), CO_2_ (3.00 ± 2.05%), NO_x_ (0.90 ± 0.32 ppm), NO (0.60 ± 0.52 ppm) were measured at the monitoring sites SP1, SP4, SP2 and SP1, respectively. PM_10_ (0.40 ± 0.52 mg/m^3^) and PM_2.5_ (0.20 ± 0.42 mg/m^3^) were observed at the highest levels in SP2. These sites were dominated by many industries, which use heavy duty generators that run on fossil fuels to power their equipment.

**Table 2:** Air pollutant concentrations at the various sampling points in Agbara industrial area.

Location codes	CO_2_ (%)	CO (ppm)	NO (ppm)	NO_X_ (ppm)	H_2_S (ppm)	VOC (ppm)	SO_2_ (ppm)	NH_3_ (ppm)	PM_2.5_ (mg/m^3^)	PM_10_ (mg/m^3^)
SP1	2.20 ± 1.03^a^	5.50 ± 2.32^a^	0.60 ± 0.52^a^	0.70 ± 0.48^a^	<0.01	<0.01	<0.01	<0.01	0.0000	0.30 ± 0.48^a^
SP2	2.10 ± 1.00^a^	4.10 ± 2.88^a^	0.30 ± 0.48^a^	0.90 ± 0.32^a^	<0.01	<0.01	<0.01	<0.01	0.20 ± 0.42^a^	0.40 ± 0.52^a^
SP3	2.00 ± 1.49^a^	1.90 ± 1.45^bc^	0.40 ± 0.52^a^	0.60 ± 0.52^a^	<0.01	<0.01	<0.01	<0.01	0.01	0.20 ± 0.42^a^
SP4	3.00 ± 2.05^a^	3.90 ± 3.11^ab^	0.50 ± 0.53^a^	0.80 ± 0.63^a^	<0.01	<0.01	<0.01	<0.01	0.01	0.10 ± 0.32^a^
SP5	2.20 ± 1.23^a^	1.10 ± 0.88^c^	0.20 ± 0.42^a^	0.67 ± 0.50^a^	<0.01	<0.01	<0.01	<0.01	0.01	0.20 ± 0.42^a^
FEPA (1991) Standards	0.1	10	0.06	0.06	0.008	3	0.2	0.28	80	
WHO (1990)				0.08						25

SP1- Industrial Roadside, SP2- Lagos Badagry express way, SP3-Market area, SP4-Banking district, SP5-Residential area, Different super scripts (alphabets) in the same column indicate significant difference at p < 0.05 according to Duncan Multiple Range Test.

It was observed that PM_2.5_ and PM_10_, CO_2_, NO and NO_x_ concentrations were all higher than the FEPA and WHO standards which poses serious health threat^19,20^. These standards were based on permissible limits for work hour exposure (8 hours) and 24 hours for particles daily exposure. The H_2_S, VOC, SO_2_ and NH_3_ values were measured below the equipment detection limit (<0.01 ppm).

It was also observed that there was no significant (P > 0.05) difference amongst the concentration of all air parameters measured except CO which had a significantly (P < 0.05) higher concentration. Furthermore, SP4 and SP2 are been dominated by heavy duty generators used by the residing manufacturing companies and banks while SP3 and SP5 had the least CO concentrations of 1.90 ± 1.45 and 1.10 ± 0.88 ppm, respectively must be attributed to the fact that the SP3 and SP5 are the market area and residential areas. The results depicted that NO, NO_x_, and PM_10_ all did not having any significant (P > 0.05) variations at the sampling sites. The presence of these pollutants at high concentrations in the atmosphere can be attributed to industrial processes such as combustion from furnace, smelting of metals, melting of glass and plastics.

Higher concentrations of CO_2_ are likely to have on global climate and profound direct effects on the growth, physiology, and chemistry of plants, independent of any effects on climate^20^. These effects result from the central importance of CO_2_ to plant metabolism. CO is a weak greenhouse gas, but its influence on climate goes beyond its own direct effects. Its presence affects concentrations of other greenhouse gases including carbon dioxide, methane and tropospheric ozone. It readily reacts with the hydroxyl radical (OH) forming a much stronger, greenhouse gas CO_2_. The finest of these particles penetrate to sensitive tissue through ingestion, inhalation or dermal contact and causing serious damages and eventually death. Inhalation may cause serious respiratory illness and diseases aggravating to heart disease^21^. Among the grievous kind of air pollutant are particulates matter due to their ability to penetrate deep into the lungs and blood streams unfiltered, causing permanent DNA mutations, cardiac attacks and subsequently death^22^. In 2013, in nine European countries study involving over three hundred thousand people reveals that the lung cancer rate rose by 22% and 36% for every increase of 10 μg/m^3^ in PM_10_ and PM_2.5_, respectively^23^.

### Dust samples

Table 3 presents the heavy metal concentrations in dust samples collected at different sampling points (mg/kg) in Agbara industrial area. It was observed that Pb, Cd, Cr, Cu and Zn concentrations were far above the FEPA and WHO permissible standards. Zn has the highest concentration (57.40 ± 13.28 ppm) at SP3, due to close proximity to two notable industries in the study area, which uses Zn as a raw material in their production processes. Cr had its highest concentration (45.36 ± 12.37 ppm) at SP1. The monitoring site SP1 was also very close to a Glass manufacturing industries that uses Cr for glass green colouration. Cr is a very powerful colourizing agent, yielding dark greenor in higher concentrations even black colour. Together with tin oxide and arsenic, it yields emerald green glass^24^. Copper had its highest concentration at the market area, SP3 (22.80 ± 17.36 ppm), which was also close to a Cable and Wire Manufacturing Company in the study area. Among the measured metals, Pb and Cd had the highest concentrations at the highway sampling station. Major sources of lead in the air are ore and metals processing and piston-engine operating on leaded fuel. Other sources are waste incinerators, utilities, and lead-acid battery manufacturers. The nearby Plastic industry could also have contributed to high Cd concentrations in dust samples as Cd is also been used as a stabilizer in plastics^19^. Metals in dust samples were measured at the lowest concentrations at the residential area of the study area.

**Table 3:** Heavy metal concentrations in dust samples.

Location	Pb (mg/kg)	Cd (mg/kg)	Cr (mg/kg)	Cu (mg/kg)	Zn (mg/kg)
SP1	9.00 ± 4.15^a^	0.17 ± 0.14^bc^	45.36 ± 12.37^a^	17.76 ± 1.82^ab^	32.43 ± 18.48^b^
SP2	12.33 ± 4.08^a^	0.49 ± 0.60^a^	30.37 ± 14.55^bc^	18.70 ± 26.34^ab^	26.31 ± 8.49^b^
SP3	10.79 ± 4.56^a^	0.32 ± 0.36^ab^	26.41 ± 12.93^c^	22.80 ± 17.36^a^	57.40 ± 13.28^a^
SP4	11.10 ± 3.03^a^	<0.01	39.40 ± 8.53^ab^	15.30 ± 4.24^ab^	33.40 ± 14.70^b^
SP5	9.40 ± 2.12^a^	<0.01	9.70 ± 3.09^d^	6.70 ± 2.54^b^	8.80 ± 2.30^c^
FEPA (1991) Standard	0.01	0.003	2	1	3
WHO (1984) Standards	0.05	0.005	0.05	0.05	1.5

SP1- Industrial Roadside, SP2- Lagos Badagry express way, SP3-Market area, SP4-Banking district, SP5-Residential area, Different super scripts (alphabets) in the same column indicate significant difference at p < 0.05 according to Duncan Multiple Range Test.

The order of abundance of heavy metals in dust samples are in the order: Zn > Cr > Cu > Pb > Cd. The data showed that lead concentration showed no significance (P > 0.05) at five (5) sampling points. The sources are either from generator exhaust, automobile exhaust or industrial chimneys. Zinc, chromium, copper and cadmium concentration were all significantly (P < 0.05) different from one another, because each sampling point had different emission sources that are unique to each sampling points.

### Plant sample

The heavy metal concentrations in plant samples (*Heteropogon contortus* (L.) collected from the study area are presented in Table 4. The order of the heavy metal uptake by the plant samples were Cu > Zn > Pb >Cr > Cd. This is because copper is more available for plant absorption due to the addition of organic matter. The presence of humic substances could favour the absorption of heavy metals and competitions among them are well established^25^. Studies of the competition for the absorption sites and type of ligand model of cadmium, copper and zinc showed that copper had a greater binding affinity than the other two metals^25,26^. Bioavailability is influenced by physical factors such as temperature, adsorption, phase association and sequestration and can also be affected by chemical factors that influence speciation at thermodynamic equilibrium, lipid solubility, complexation kinetics, and octanol/water partition coefficients^27^. Biological factors such as species characteristics, trophic interactions, and biochemical/physiological adaptation, also play an important role^28^.

**Table 4:** Heavy metals concentration in plant samples.

Locations	Pb (mg/kg)	Cd (mg/kg)	Cr (mg/kg)	Cu (mg/kg)	Zn (mg/kg)
SP1	7.20 ± 1.77^c^	0.45 ± 0.14^b^	22.46 ± 9.35^a^	70.07 ± 16.24^a^	21.80 ± 7.51^c^
SP2	10.98 ± 1.77^b^	1.51 ± 0.37^ab^	7.75 ± 3.84^c^	54.56 ± 13.82^bc^	22.60 ± 6,03^c^
SP3	13.76 ± 3.08^a^	2.25 ± 3.04^a^	12.61 ± 2.14^b^	62.55 ± 6.90^ab^	67.69 ± 14.50^a^
SP4	4.15 ± 1.34^d^	0.26 ± 0.25^b^	8.35 ± 1.48^c^	48.01 ± 12.37^c^	36.40 ± 7.44^b^
SP5	7.36 ± 1.39^c^	0.23 ± 0.34^b^	2.88 ± 1.14^d^	28.56 ± 6.07^d^	43.40 ± 7.31^b^
WHO limits of Heavy Metals in Plants	2	0.02	1.3	10	

SP1- Industrial Roadside, SP2- Lagos Badagry express way, SP3-Market area, SP4-Banking district, SP5-Residential area, Different super scripts (alphabets) in the same column indicate significant difference at p < 0.05 according to Duncan Multiple Range Test.

### Principal Component Analysis (PCA)

#### Air sample analysis

Table 5 shows the rotated PCA data of the air pollutants. The essence of PCA model is to identify the possible sources of measured air pollutants. Three factors were identified with 65% of dataset explained. Factor1 with highest % variance of 27% has high positive loadings for CO, NO and NOx. This source indicates vehicular emission from mobile combustion of fossil fuel in line with published work^29^. Factor 2 has a significant affinity for CO_2_ and PM_2.5_ and can be designated an industrial source. Factor 3 was only significant for PM_10_, the possible emission source might be from road dust re-suspension^22^.

**Table 5:** Principal component analysis of air pollutants.

Parameters	Factors			Communalities
	**1**	**2**	**3**	
CO_2_	−0.013	**0.698**	0.156	**0.511**
CO	**0.623**	0.010	0.158	**0.414**
NO	**0.757**	−0.226	−0.287	**0.706**
NO_X_	**0.819**	0.115	0.046	**0.686**
PM_10_	0.042	−0.025	**0.954**	**0.913**
PM_2.5_	−0.003	**0.818**	−0.205	**0.711**
%Variance	27	20	18	
Sources	Vehicular	Industrial 1	Road dust	

#### Dust analysis

Table 6 presents the rotated PCA data of the metals in dust samples. Two factors were identified by the multivariate model explaining 66% of the total data variance. Factor 1 has high loadings for Pb, Cd and Zn while Factor 2 was highly loaded for Cr and Cu. The prominent emission sources were industrial and traffic, respectively. Taiwo *et al*.^30,31^ had shown steel industry as major emitters of Pb, Cd and Zn, while Cu had been adopted as a signature for vehicular brake dust.

**Table 6:** Principal component analysis of metals in dusts.

Parameters	Factors		Communalities
	1	2	
Pb	**0.841**	0.153	**0.731**
Cd	**0.653**	0.221	**0.475**
Cr	−0.022	**0.813**	**0.662**
Cu	0.146	**0.828**	**0.707**
Zn	**0.775**	−0.340	**0.716**
%Variance	35	31	
Sources	Engine exhaust	Industrial	

The SPE-risk model is shown in Table 7. The model indicates that carbon dioxide and carbon monoxide have the greatest health risk, which needs to be regulated urgently. The major sources of these gaseous pollutants as indicated by the PCA model were industrial and vehicular emissions. The next health threat, which fell on the substantial range, was PM_2.5_ and PM_10_. These particulate pollutants originated from various sources and are accumulated along the major road networks as soil and road dust. Such sources include road re-suspended dust, industrial emission, construction materials used (sand and cement), sooth from cooking stoves, charcoal or generators which are deposited by wind to the high way. The next are nitrogen monoxide, oxides of nitrogen, volatile organic compounds and Sulphur dioxide that posed a significantly lower health threat due to lower exposure rate and lower concentration. The least health risks were observed for ammonia and hydrogen sulphide, probably due to their very low concentration in the area and weaker effects to human health. The SPE model of heavy metals indicated that copper, zinc and lead have the highest health risks, owing to their very high concentrations in the vicinity (Table 8). The least health threat was measured for cadmium because of its extremely lower concentration and low exposure rate.

**Table 7:** Risk analysis of air pollutants on the environment and human at Agbara industrial district.

Parameter Measured	F. Identity Risk	Probability (P)	Severity (S)	Exposure (E)	Risk Calculation (SxPxE)	Risk Level	Risk
Carbon dioxide (CO_2_)concentration at Agbara industrial district	Exposure to its on-site concentration	Frequent (5)	Critical (3)	Great (4)	60.00	High	Immediate correction needed before proceeding. Worn-out control devices at industries should be replaced while VIO should be more proactive with vehicle emission inspection. Continuous oversight & re-evaluation is required. Risk controls must reduce the level of risk to one that is lower than initially calculated.
Carbon monoxide (CO) concentration at Agbara industrial district	Exposure to its on-site concentration	Frequent (5)	Catastrophic (4)	Above Average (3)	60.00	High	Immediate correction needed before proceeding. Worn-out control devices at industries should be replaced while VIO should be more proactive with vehicle emission inspection. Continuous oversight & re-evaluation is required. Risk controls must reduce the level of risk to one that is lower than initially calculated.
Nitrogen oxide (NO) concentration at Agbara industrial district	Exposure to its on-site concentration	Likely (4)	Critical (3)	Average (2)	24.00	Possible	Acceptable. Some attention needed. Implement risk reduction controls to further reduce the level of risk as appropriate.
Other oxides of nitrogen (NOx) concentration at Agbara industrial district	Exposure to its on-site concentration	Likely (4)	Critical (3)	Average (2)	24.00	Possible	Acceptable. Some attention needed. Implement risk reduction controls to further reduce the level of risk as appropriate.
Hydrogen sulphide (H_2_S) concentration at Agbara industrial district	Exposure to its on-site concentration	Rarely (1)	Minor (1)	Below average (1)	1.00	Slight	Acceptable
Volatile organic compounds (VOC) concentration at Agbara industrial district	Exposure to its on-site concentration	Occasional (3)	Catastrophic (4)	Average (2)	24.00	Possible	Acceptable. Some attention needed. Implement risk reduction controls to further reduce the level of risk as appropriate.
Ammonia (NH3) concentration at Agbara industrial district	Exposure to its on-site concentration	Likely (4)	Minor (1)	Above Average (3)	12.00	Slight	Acceptable
Sulphur dioxide (SO2) concentration at Agbara industrial district	Exposure to its on-site concentration	Occasional (3)	Significant (4)	Above Average (3)	36.00	Possible	Acceptable. Some attention needed. Implement risk reduction controls to further reduce the level of risk as appropriate.
Particulate matter (PM2.5) concentration at Agbara industrial district	Exposure to its on-site concentration	Frequent (5)	Critical (3)	Above Average (3)	45.00	Substantial	Correction and/or risk mitigation is required. Mitigation controls have to be implemented. Risk controls must reduce the level of risk to one that is lower than was initially calculated.
Particulate matter (PM10) concentration at Agbara industrial district	Exposure to its on-site concentration	Frequent (5)	Critical (3)	Above Average (3)	45.00	Substantial	Correction and/or risk mitigation is required. Mitigation controls have to be implemented. Risk controls must reduce the level of risk to one that is lower than was initially calculated.

**Table 8:** Risk analysis of heavy metals in dust on the environment and human at Agbara industrial district.

Parameter Measured	F. Identity Risk	Probability (P)	Severity (S)	Exposure (E)	Risk Calculation (SxPxE)	Risk Level	Risk
Lead concentration at Agbara industrial district	Exposure to its on-site concentration	Likely (4)	Catastrophic (4)	Significant (4)	64.00	High	Immediate correction needed before proceeding. Worn-out control devices at industries should be replaced while VIO should be more proactive with vehicle emission inspection. Continuous oversight & re-evaluation is required. Risk controls must reduce the level of risk to one that is lower than initially calculated.
Chromium concentration at Agbara industrial district	Exposure to its on-site concentration	Occasional (3)	Critical (3)	Significant (4)	36.00	Possible	Acceptable. Some attention needed. Implement risk reduction controls to further reduce the level of risk as appropriate.
Cadmuim concentration at Agbara industrial district	Exposure to its on-site concentration	Rarely (2)	Catastrophic (4)	Below average (1)	8.00	Slight	Acceptable.
Copper concentration at Agbara industrial district	Exposure to its on-site concentration	Frequent (5)	Critical (3)	Great (4)	60.00	High	Immediate correction needed. Worn-out control devices at industries should be replaced. Continuous oversight & re-evaluation is required. Risk controls must reduce the level of risk to one that is lower than initially calculated.
Zinc concentration at Agbara industrial district	Exposure to its on-site concentration	Frequent (5)	Critical (3)	Significant (4)	60.00	High	Immediate correction needed. Worn-out control devices at industries should be replaced. Continuous oversight & re-evaluation is required. Risk controls must reduce the level of risk to one that is lower than initially calculated.

## Conclusion

The study had assessed the environmental pollution and related hazards of industries at Agbara, Ogun State, Nigeria. Results showed that CO_2_, NO and NOx concentrations were higher than the WHO and FEPA permissible standards for work exposure at all five sampling points. The high CO_2_ value measured in this study is of great environmental concern due to its major contributor to the green house gas and climate change. The NO and NOx are also a big threat, which are formed during extremely high temperature combustion processes. Also, Pb, Cd, Cr, Cu and Zn all had concentrations above the WHO and FEPA standards at all five sampling points. The trend of heavy metals in dust at Agbara industrial district was Zn > Cr > Cu > Pb > Cd. The concentration of heavy metals found in plants followed the sequence: Cu > Zn > Pb > Cr > Cd. The principal component analysis revealed traffic and industrial emissions as major contributors to air pollution in the study area. The SPE model revealed carbon dioxide and carbon monoxide as important air pollutants with greatest health threats in the study area. For heavy metals, the SPE model showed copper, zinc and lead as most hazardous metals with the highest health risks. This study showed industries and traffic as the major threats to urban atmospheric environment.

## References

[1] Mohammad IL, Zhen-Li H, Peter JS, Xiao-e Y (2008). Phytoremediation of heavy metal polluted soils and water: Progresses and perspectives. Journal of Zhejiang University Science.

[2] McGrath SP, Zhao FJ, Lombi E (2001). Plant and rhizosphere process involved in phytoremediation of metal-contaminated soils. Plant Soil.

[3] Garbisu C, Alkorta I (2001). Phytoextraction: A cost effective plant-based technology for the removal of metals from the environment. Biores Technology.

[4] Bickerstaff K, Walker G (2001). Public understandings of air pollution: The “localisation” of environmental risk. Global Environmental Change..

[5] Leem JH (2006). Exposures to air pollutants during pregnancy and preterm delivery. Environmental Health Perspective..

[6] World Health Organization. Air Quality and Health. Available online: http://www.who.int/mediacentre/factsheets/fs313/en/ (17 Sept. 2014) (2013).

[7] Bell ML (2005). Challenges and recommendations for the study of socioeconomic factors and air pollution health effects. Environmental Science Policy..

[8] World Health Organization 7 million premature deaths annually linked to air pollution. Retrieved. pp. 85–87 (2015).

[9] Ibeto, C., Okoye, C., Ofoefule, A. & Uzodinma, E. Analysis of Environmental Pollutants by Atomic Absorption Spectrophotometry, Macro to NanoSpectroscopy, http://www.intechopen.com/books/macro-tonanospectroscopy/analysis-ofenvironmental-pollutants-by-atomic-absorption-spectrophotometry (7 Feb. 2014) (2012).

[10] Olayinka OO, Adedeji OH, Oladeru IB (2013). Water Quality and BacteriologicalAssessment of Slaughterhouse Effluent on Urban River in Nigeria. Journal of Applied Sciences in Environmental Sanitation.

[11] Ibeto CN, Okoye COB (2010). Elevated Levels of Lead in Blood of Different Groupsin the Urban Population of Enugu State. Nigeria.International Journal of Human and Ecological Risk Assessment..

[12] Zhang MK, Dang Z, Zheng LC, Yi XY (2010). Remediation of soil co-contaminated with pyrene and cadmium by growing maize (Zea mays L*.)*. International Journal of Environmental Science and Technology..

[13] GWRTAC. Remediation of metals-contaminated soils and groundwater, Tech. Rep. TE-97-01, GWRTAC, Pittsburgh, Pa, USA, GWRTAC-E Series (1997).

[14] Kirpichtchikova TA (2006). Speciation and solubility of heavy metals in contaminated soil using X-ray microfluorescence, EXAFS spectroscopy, chemical extraction, and thermodynamic modeling. Geochimica et Cosmochimica Acta.

[15] Adriano, D. C. Trace Elements in Terrestrial Environments: Biogeochemistry, Bioavailability and Risks of Metals, Springer, New York, NY, USA, 2nd edition (2003).

[16] Maslin P, Maier RM (2000). Rhamnolipid-enhanced mineralization of phenanthrene in organic-metal co-contaminated soils. Bioremediation Journal.

[17] McLaughlin MJ, Hamon RE, McLaren RG, Speir TW, Rogers SL (2000). Review: a bioavailability-based rationale for controlling metal and metalloid contamination of agricultural land in Australia and New Zealand. Australian Journal of Soil Research..

[18] Bodunrin JO, Ajayi OS (2017). Natural Radioactivity Measurements To Determine The Radiation Hazards From Surface Soil And Effluents In Agbara Industrial Estate, Ogun State, Nigeria. International Journal of Innovative Research and Advanced Studies (IJIRAS).

[19] WHO. Water sanitation hygiene. The World Health Organization, Geneva, http://www.who.int/water_sanitation_health/dwq/GDW12rev1and2.pdf. Accessed 24/02/2018(2006).

[20] FEPA. Nigerian Standard on Environmental Parameters and Control. Nigeria. A manual guide for environmental usage (1991).

[21] Zhang MK, Dang Z, Zheng LC, Yi XY (2009). Remediation of soil co-contaminated with pyrene and cadmium by growing maize (Zea mays L.). International Journal of Environmental Science and Technology.

[22] USEPA. Treatment technologies for site cleanup: annual status report (12th Edition),” Tech. Rep. EPA-542-R-07-012, Solid Waste and Emergency Response (5203P), Washington, DC, USA. (2008).

[23] Taiwo AM, Harrison RM, Shi Z (2014). A review of receptor modelling of industrially emitted particulate matter. Atmospheric environment.

[24] Ole RN (2013). Air pollution and lung cancer incidence in 17 European Cohorts; perspective analyses from European study of Cohorts for air pollution effect (ESCAPE). Land Oncology.

[25] Bode. P (1993). The use of INAA for determination of trace elements, in particular cadmium, in plastics in relation to the enforcement of pollution standards. Journal of Radioanalytical and nuclear chemistry of Netherlands..

[26] Navarror-Pedreno, J., Almendro-Candel, M.B., Jordan, M.M., Mataix-Solera, J., Garcia-Sanchez. E. Risk areas in the application of sewage sludge on degraded soils in the province of Alicante (Spain). Geo-Environment: Monitoring and remediation of the geological environment 2004: 293–302 (2004).

[27] Ramalho CT, Figueroa-Villar DJ (2002). Thermodynamic evaluation of complexes of zinc and cadmium that mimetize metallic centers in transcription factors. Journal of Molecular Structure THEOCHEM..

[28] Hamelink, J. L., Landrum, P.F., Harold, B.L., William, B.H. Bioavailability: Physical, Chemical, and Biological Interactions. Boca Raton, FL: CRC Press Inc (1994).

[29] Verkleji, J.A.S. The effects of heavy metals stress on higher plants and their use as biomonitors In Plant as Bioindicators: Indicators of Heavy Metals in the Terrestrial Environment. Markert B, editor. New York: VCH (1993).

[30] Hao J, Wu Y, Fu L, He D, He K (2001). Source contributions to ambient concentrations of CO and NOx in the urban area of Beijing. Journal of Environmental Science and Health, Part A.

[31] Taiwo AM (2014). Receptor modelling of airborne particulate matter in the vicinity of a major steelworks site. Science of the Total Environment.

